# Efficient Hepatic Delivery of Drugs: Novel Strategies and Their Significance

**DOI:** 10.1155/2013/382184

**Published:** 2013-10-28

**Authors:** Nidhi Mishra, Narayan Prasad Yadav, Vineet Kumar Rai, Priyam Sinha, Kuldeep Singh Yadav, Sanyog Jain, Sumit Arora

**Affiliations:** ^1^Herbal Medicinal Products Department, CSIR-Central Institute of Medicinal and Aromatic Plants (CSIR-CIMAP), Lucknow 226015, India; ^2^Centre of Pharmaceutical Nanotechnology, Department of Pharmaceutics, National Institute of Pharmaceutical Education and Research (NIPER), Sector 67, SAS Nagar, Mohali, Punjab 160062, India

## Abstract

Liver is a vital organ responsible for plethora of functions including detoxification, protein synthesis, and the production of biochemicals necessary for the sustenance of life. Therefore, patients with chronic liver diseases such as viral hepatitis, liver cirrhosis, and hepatocellular carcinoma need immediate attention to sustain life and as a result are often exposed to the prolonged treatment with drugs/herbal medications. Lack of site-specific delivery of these medications to the hepatocytes/nonparenchymal cells and adverse effects associated with their off-target interactions limit their continuous use. This calls for the development and fabrication of targeted delivery systems which can deliver the drug payload at the desired site of action for defined period of time. The primary aim of drug targeting is to manipulate the whole body distribution of drugs, that is, to prevent distribution to non-target cells and concomitantly increase the drug concentration at the targeted site. Carrier molecules are designed for their selective cellular uptake, taking advantage of specific receptors or binding sites present on the surface membrane of the target cell. In this review, various aspects of liver targeting of drug molecules and herbal medications have been discussed which elucidate the importance of delivering the drugs/herbal medications at their desired site of action.

## 1. Introduction

In the last decade, significant advances have been made in the development of plant-based hepatoprotective drugs mostly because of their lower toxicity and a multi-factorial approach to restoring health, seeking equilibrium between mind, body, and environment and placing a greater emphasis on the multidimensional elements of health than on pathology alone. Along with drugs, phytomedications have increasingly been prescribed for the treatment of a number of diseases. However, phytotherapeutics needs a scientific approach to deliver the components in a sustained manner so as to increase patient compliance and avoid repeated administration. This can be achieved by designing novel drug delivery systems (NDDS) for herbal constituents, in addition to the drugs already available in the market. Novel drug delivery systems not only reduce the repeated administration (due to its sustained-release properties) to overcome noncompliance but also help to increase the therapeutic value by reducing toxicity, increasing the bioavailability, stability, and targetability to a specific cell or organ (due to its subcellular size). For a long time, herbal medicines were not considered for development as novel formulations owing to the lack of scientific justification and processing difficulties, such as standardization, extraction, and identification of individual drug components in complex polyherbal systems. However, modern phytopharmaceutical research solves the scientific needs for herbal medicines as in modern medicine, which gives way for developing novel formulations such as nanoparticles, microemulsions, matrix systems, solid dispersions, liposomes, and solid lipid nanoparticles.

However, for delivery to specific cell type of liver, novel drugs delivery system for herbal drugs, still needs some modification such as attaching of ligand or targeting moiety which will recognize and interact with specific cell type of liver. In the present review, we enumerate all the methods for attaching targeting moiety to delivery system and different factors which could be taken into account while designing NDDS for liver cell which will be of immense importance in near future. The review elucidates the importance of delivery of both the drugs and herbal medications to the liver so as to ensure successful treatment outcomes. 

## 2. Morphological Study of Liver 

Before discussing the different methods of targeting, it is necessary to understand the morphology of liver (especially vascular supply) and molecular scale of the target tissue in order to design a novel drug delivery system rationally.

The liver engages numerous metabolic, immunological, and endocrine functions. It receives blood (oxygenated and deoxygenated) from the gut and heart via the portal vein and hepatic artery, respectively. Blood circulates through a permeable discontinuous capillary network term as the *sinusoids* to reach the central and hepatic veins. The sinusoids are small blood vessels (5 to 10 *μ*m wide) between the radiating rows of hepatocytes having fenestrations of size 100–150 nm (depending on the type of animal species). They allow almost unrestricted passage of plasma components to the *perisinusoidal space*, where the cords of parenchymal cells called as hepatocytes are situated. Inside the sinusoid capillaries, the *Kupffer cells* are responsible for phagocytic activity of the liver [1, 2].

### 2.1. Phagocytosis in Kupffer Cells

Phagocytosis occurs after the multivalent drug delivery system comes in contact with the macrophage where they spread the cell membrane around the particles to engulf them. Macrophages recognize the delivery systems via the recognition of opsonins present over them or through interaction with scavenger receptors present on Kupffer cells. After ingestion, phagocytic vesicle (phagosomes) coalesces with intracellular organelles containing digestive proteins having acidic internal pH, to mature into phagolysosomes and to degrade the internal part of the delivery system. The delivery system is then eliminated by exocytosis after degradation or is sequestered in residual bodies within the cell if it cannot be digested [3, 4].

### 2.2. Macrophages Interaction with the Delivery System

The following factors should be taken into account while discussing the interaction of macrophages with the delivery system.

*Size and Radius.* The interaction between the macrophages and delivery system could be influenced by the size and radius of curvature of delivery system. Generally, a diameter of 1–3 *μ*m (*in vitro *limit is 20 *μ*m) is sufficient for interaction as Kupffer cell has ruffled surface. Too small particles co-operate less with the cell membrane and gain entry into the cell through the other side like pinocytosis or endocytosis while the larger particle fails to contact with the cell membrane.

*Shape.* The shape has great impact on the interaction of delivery system and macrophages. For larger particles, elongated shapes promote interaction while for smaller particle shape influences the speed of internalization and the different pathways used to enter the cells. Some studies have shown that, in comparison to their spherical shape, nonspherical particles are steered into distinctive tissue distribution patterns by hydrodynamic forces in the bloodstream, for example, filomicelles with very high aspect ratios (>10) and longitudinal lengths around 10 *μ*m achieve considerably longer circulation times than spherical particles.

*Flexibility and Deformability.* These are other parameters that also influence the interaction and distribution. The flexibility of smaller hydrophilic delivery system (approximately 200 nm) was found to affect *in vitro* kinetics and internalization pathways in macrophages [5] and other nonphagocytic cells [6]. *In vivo*, the flexibility and deformability of delivery systems have great impact on their tissue distribution and retention, for example, RBC.

**Figure 1:**
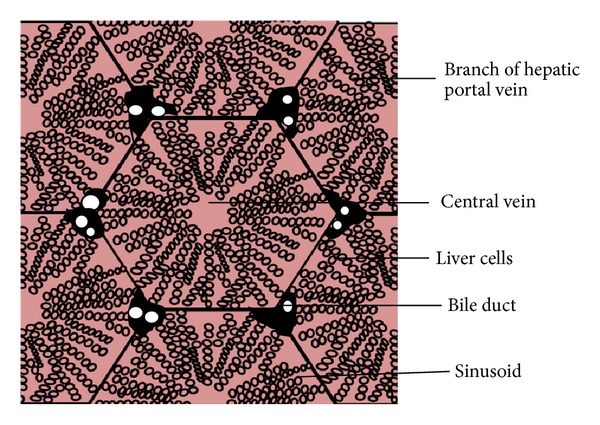
Microscopic view of liver cells.

*Surface Properties.* These properties influence interaction in a complex manner. Positive charges on the surface of delivery system have deleterious effect on circulation times, whereas contrary findings are reported regarding the impact of negative charge. For example, He et al. [7] studied the effects of particle size and surface charge on cellular uptake/biodistribution and concluded that *in vivo* biodistribution of nanoparticles (NPs) with slight negative charges and particle size of 150 nm tended to accumulate in tumor more efficiently, while Funato et al. and Nishikawa et al. [8, 9] reported that negatively charged liposomes have a shorter half-life in the blood.  Figure 1 provides a microscopic view of the liver surface.

## 3. Essential Attributes for Designing Delivery Systems for Liver Targeting

For a therapeutic moiety to exert its desired effect, it needs to be in physical contact with its physiological target such as a receptor present on liver cells. Site-specific drug delivery ensures that such interactions take place only in the desired anatomical location of the liver; therefore, it must fulfil the following criteria: (i) it must be able to cross the anatomical barriers such as those of stomach and intestine, (ii) should be recognized selectively by the receptor present on liver cell such as asialoglycoprotein, (iii) exogenously delivered ligand for targeting should compete with the endogenously produced ligand, (iv) fabricated delivery system must be nontoxic, biocompatible, biodegradable, and physico-chemically stable in the liver cells either *in vivo *or* in vitro*, (v) it should have uniform sinusoid capillary distribution, (vi) should have controllable and predictable rate of drug release so that only therapeutic amount of drug is released to the liver cells, (vii) drug release should not affect the drug distribution, (viii) it should show minimal drug leakage during its passage through stomach, intestine, and other parts of the body, (ix) carrier used for encapsulating the herbal drugs must be eliminated from the body without imparting any sign of toxicity and no carrier should induce modulation of diseased state, and (x) lastly, preparation of drug delivery system should be easy or reasonably simple, reproducible, and cost-effective.

### 3.1. Pharmacokinetic Considerations

Pharmacokinetics also plays an important role in developing novel delivery systems of herbal drugs for liver, since the introduction of this tool enables us to quantitatively predict the disposition of drugs after modification. Some pharmacokinetic conditions must be fulfilled, to achieve a successful performance by site-selective drug-carrier delivery systems which are as follows.

#### 3.1.1. Rate of Elimination of Drug-Carrier Conjugate

Drug-carrier conjugate should not be removed too rapidly from the systemic circulation rather should be removed in a controlled manner. All the nonspecific interactions between drug-carrier conjugate and the environment of the systemic compartment need to be eliminated during designing and development of targeted delivery systems. The carrier should have the ability to restrict all unwanted interactions between the drug and the physiological environment until drug is released at the target site [10, 11].

#### 3.1.2. Rate of Release of Free Drug at the Non-Target Site

The release of drug at the non-target site could nullify any benefits that might potentially come from delivering the drug to the target site. This is because the amount of drug reaching at the nonspecific sites may cause toxicity owing to its high concentration. 

#### 3.1.3. Rate of Delivery of Drug-Carrier Conjugate to the Target Site

If the drug-carrier conjugate reaches the target site too slowly, the supply of free drug might never be sufficient to generate the concentration required to elicit the desired therapeutic effect at the site of action. The total amount of drug delivered (i.e., the area under the curve in a drug concentration versus time plot for the target site) is irrelevant if, at any time, the free-drug concentration at the target site does not reach its pharmacologically effective level. Delivery of the drug-carrier conjugate to the target organ may not guarantee that an adequate amount of the free drug will be available at the actual target.

#### 3.1.4. Rate of Release of Free Drug at the Target Site

The capacity of the drug delivery system selected for the release of free drug from the conjugate should be considered. It needs to be suitable for processing the entirety of the drug-carrier conjugate arriving at the target site, doing so at a rate that also ensures drug accumulation at target site.

#### 3.1.5. Rate of Removal of Free Drug from the Target Site

Drugs that benefit most from target selective delivery are those that are retained at the site while acting on their target of action. Therefore, drugs should be specifically designed to be used with target selective delivery systems and drug delivery should not be used for rescuing poorly performing, existing drugs. Certain drug (e.g., DNA in gene therapy) needs to be delivered into the cytoplasm; therefore, it would be preferential for the release of the drug to take place within the cells. This could lead to the enhanced retention of the drug in close proximity to its target. Furthermore, an increased rate of elimination of free drug from the central compartment tends to increase the advantage brought about as a result of drug targeting but also increases the required rate of input (of the drug carrier) to maintain a therapeutic effect [12].

#### 3.1.6. Rate of Elimination of the Drug-Carrier Conjugate and Free Drug from the Body

For optimal targeting, elimination of the complete drug-carrier system should be minimal. These systems are too large to be eliminated via the kidneys [13]. Consequently, the liver is mainly responsible for the removal of drug conjugates from the circulation. The rate of elimination of free drug from the systemic circulation should be rapid relative to its rate of transfer from the target site to the central compartment of the body. This way, the drug-delivery system will achieve a decrease in the drug-associated toxicity.

## 4. Formulation Aspects of Liver Targeting of Drugs 

Normally most of the drugs achieve high hepatic concentration, still their targeting is necessary because liver is the major organ in the body equipped for uptake, detoxification, metabolic transformation, and excretion of xenobiotics into bile by means of carrier-mediated mechanism. As a consequence, most of the drugs are rapidly cleared from the blood and display high first pass clearances by the liver. However, it should be realized that the total hepatic uptake predominantly depends on hepatocytes, whereas Kupffer cells largely contribute to hepatic uptake of particulate material. Therefore, the drugs that enter the liver as such or in the form of covalent carrier conjugates will not necessarily reach the required cell type. Moreover, if drugs are accumulated in the liver, their residence time in the organ is influenced by the factors discussed under macrophages interaction with delivery system and pharmacokinetic consideration. Thus, the challenge is to obtain selective accumulation of drugs in one specific cell type and to sustain intracellular levels for longer period. The target cells/receptors within the liver for treatment and possible entry mechanisms in these cells have been identified (Table 1). Hepatocytes are functional units responsible for most of the metabolic and secretory activities of the liver. Small size delivery system, that is, 150 nm, avoid capture by Kupffer cells and can diffuse out of the sinusoids through the fenestrations and reach the hepatocytes plates. These cells can take up colloidal carrier system through pinocytosis and receptor-mediated endocytosis. Improved delivery (enhanced localization) to the parenchyma is achieved with small size delivery system, that is, ≤50 nm, that can diffuse deeper in the space of disse [2, 14, 15]. Specific targeting of hepatocyte receptors can also be achieved. The most commonly exploited target is the asialoglycoprotein receptor (ASGP-R) that recognizes carbohydrates (mainly galactose and N-acetylgalactosamine) with variable affinity [16]. Since ASGP-R positive vesicles transit to the lysosomes, the increasingly acidic and oxidative conditions in organelles after endocytosis must be taken into account when targeting this pathway. Similarly, the upper size threshold for the internalization of colloids via ASGP-R seems to be situated below 90 nm [17]. This limits the type of carrier system which can be delivered through this route. Another approach consists of decorating delivery system with hepatocytes targeting lipoproteins either before administration [18, 19] or *in situ* after injection [20, 21]. Although effective targeting to hepatocytes can be achieved, the ubiquitous nature of lipoprotein receptors in different tissues can lead to nonspecific distribution and subsequent side effects [22]. Finally, because non-parenchymal liver cells also possess physiological and pathological functions, their targeting can sometimes be desirable. Scavenging receptors can serve to target Kupffer and endothelial cells [21, 23], while coupling with vitamin A on the surface of colloidal delivery system revealed an effective way of delivering active to stellate cells that play a fundamental role in liver fibrosis [24].

### 4.1. Different Aspects of Formulation Design for Targeting to Liver

**Figure 2:**
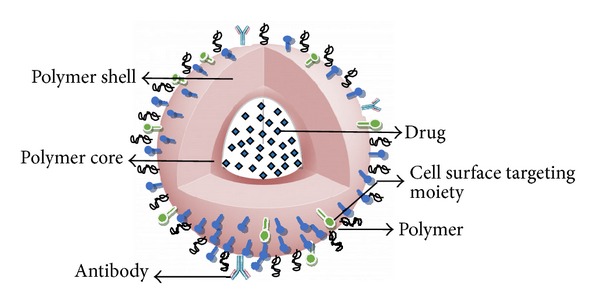
Drug delivery system encapsulating drug grafted with targeting moiety.

There are various methods for coupling of ligand molecule on drug delivery system so that drug-carrier system can be targeted to liver cell via receptor ligand processes (Figure 2). Some of them are discussed below and which can also be used for targeting of herbal drugs to liver.

**Figure 3:**
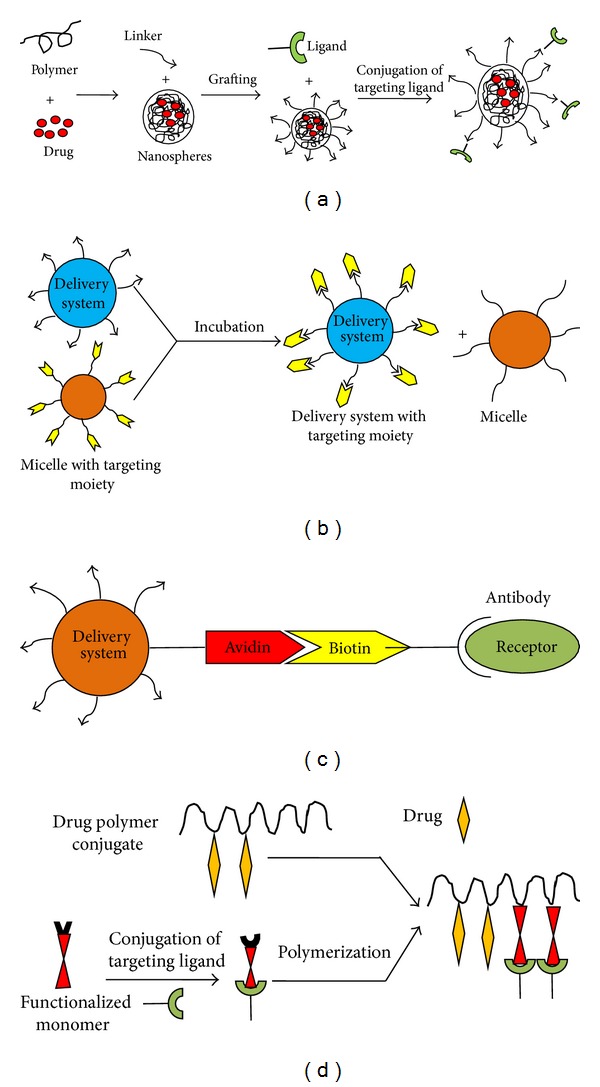
Different methods for coupling of ligand molecule on drug delivery system: (a) Coupling of targeting moieties on preformed nanocarriers. (b) Coupling of targeting moieties by the post insertion method. (c) Coupling of targeting moieties by the Avidin/Biotin complex. (d) Coupling of targeting moieties before nanocarriers formulation.

Different methods for coupling of ligand molecule on drug delivery system (Figure 3) are

- coupling of targeting moieties on preformed nanocarriers,
- coupling of targeting moieties by the post-insertion method,
- coupling of targeting moieties by the Avidin/Biotin complex,
- coupling of targeting moieties before nanocarriers formulation.

#### 4.1.1. Coupling of Targeting Moieties on Preformed Nanocarriers

The design of nanocarriers possessing targeting moieties on their surface can be realized by coupling of the selected molecule to the surface of preformed nanocarriers using various methods of the coupling chemistry domain. Considerable amount of work has been done on the coupling of antibodies on the surface of preformed nanocarriers using maleimide groups located on their surface. Sugars have been also widely used as targeting moieties. Liang et al. [26] have prepared nanoparticles composed of (PGA-PLA) processing galactosamide on their surfaces. Cellular uptake study, using rhodamine-123 loaded PGA-PLA nanoparticle with conjugated galactosamine, indicated that galactosylated nanoparticles had a specific interaction with HepG2 cells via ligand-receptor (ASGP-R) recognition. Viability of HepG2 cells treated with different paclitaxel formulations showed that the activity inhibiting the growth of cells by paclitaxel loaded galactosylated PGA-PLA nanoparticles was comparable to that of clinically available paclitaxel (Phyxol@) while paclitaxel loaded PGA-PLA nanoparticles displayed a significantly lower activity. The authors concluded that the galactosylated nanoparticles interacted in a specific manner with HepG2 cells via a ligand-receptor (ASGP-R) recognition leading to internalization of the drug carrier into HepG2 cells and release of paclitaxel into the cytoplasm. Biodistributions of the prepared nanoparticles in organs of normal mice and hepatoma bearing nude mice showed that galactosylated nanoparticles had specific interactions with liver's parenchymal cells and HepG2 cells via ligand receptor recognition. In addition, antitumor efficacy of the prepared nanoparticles on hepatoma bearing nude mice showed that paclitaxel loaded galactosylated PGA-PLA nanoparticles have the higher efficacy in reducing the tumor size. The results led the authors to conclude that paclitaxel (TX) loaded galactosylated PGA-PLA nanoparticles were mainly accumulated at the tumor site and the liver, in contrast to a nonspecific accumulation of Phyxol@.

Gupta et al. [27] reported that PLGA nanoparticles bearing HBsAg were prepared by double emulsion method and furthermore lectin from *Arachis hypogaea* (peanut agglutinin) was anchored onto the surface of the HBsAg loaded nanoparticles in order to enhance their affinity towards the antigen presenting cells of the Peyer's patches. They concluded that the ligand-coupled nanoparticles demonstrated approximately fourfolds increase in degree of interaction with the bovine submaxillary mucin (BSM) as compared to plain nanoparticles.

In another study, Bibby et al. [28] reported the bio-distribution and pharmacokinetics of a cyclic RGD-doxorubicin-nanoparticle (NP) formulation in tumor-bearing mice. The NP core was composed of insulin multimethacrylate with a targeting peptide, cyclic RGD, covalently attached to the NPs via PEG-400 and revealed decreasing drug concentrations over time in the heart, lung, kidney, and plasma and accumulating drug concentrations in the liver, spleen, and tumor due to drug-receptor interaction as particle largely composed of carbohydrate, the insulin derivative, that is, insulin multimethacrylate (IMMA).

Stella et al. [29] studied the design of poly(ethylene glycol) (PEG)-coated biodegradable nanoparticles coupled to folic acid to target the folate-binding protein. In this study, preformed nanoparticles were conjugated to the activated folic acid via PEG terminal amino groups and purified from unreacted products and the authors concluded that folate-linked nanoparticles represent a potential new drug carrier for tumor cell-selective targeting.

Coupling of a targeting moiety on the surface of preformed nanocarriers has brought a significant improvement in the designing of targeted drug delivery systems.

#### 4.1.2. Coupling of Targeting Moieties by the Post-Insertion Method

The modifications of preformed nanocarriers do not always lead to a controlled amount of bound targeting moieties, so other ways of coupling have been studied and the most important one is post-insertion method. The post-insertion technique seems to be relatively simple, leads to an appropriate level of stable ligand incorporation, and is not compromising for drug loading efficacy and drug release profile [30]. The post-insertion consists of firstly preparing the drug delivery system loaded with the selected drug, parallely micelles based on a mixture of functionalized PEG-lipid are prepared, and the selected targeting moiety is coupled to it. Secondly, the targeting moiety is transferred from micelles to drug delivery system by incubating both formulations. The post-insertion technique seems to be simple and leads to the expected site specific drug nanocarriers. However, number of targeting moieties on carrier surface was not always well defined and a drug leakage was observed during the incubation procedure.

Iden and Allen [31] prepared the stealth immunoliposomes (SIL) coupled to anti-CD19 by the post-insertion technique and revealed that the *in vitro* binding and uptake of PIL[anti-CD19] by CD19-expressing B-cell lymphoma (Namalwa) cells were similar to those of SIL[anti-CD19] and both were significantly higher than binding of non-targeted liposomes (SL). In addition, no significant differences were found between the respective *in vitro* cytotoxicities of doxorubicin-loaded PIL [anti-CD19] or SIL[anti-CD19], or in their *in vivo* therapeutic efficacy in a murine model of human B-lymphoma. Overall, the results demonstrate that the post-insertion technique is simple, flexible and effective means for preparing targeted liposomal formulations for clinical applications.

#### 4.1.3. Coupling of Targeting Moieties by the Avidin/Biotin Complex

Avidin is a basic glycoprotein (MW 68 Kd) which has a high affinity for the small (MW 24 Kd) water soluble vitamin biotin. Biotin can be conjugated to a variety of biological molecules, including antibodies, and many biotin molecules can be attached to a single molecule of protein. The biotinylated protein can thus bind to more than one molecule of avidin. The strong avidin-biotin complex has been used to couple targeting moieties on nanocarrier's surfaces with the advantage that no coupling chemistry is normally needed. Bio-distribution of lactosyl-streptavidin (Lac-St, 5 mol Lac/mol St), examined during period of several days, showed rapid uptake by the liver and almost none by blood and other tissues. Avidin and streptavidin were further subjected to an array of chemical modifications in an attempt to identify other tissue specificities. Chemical modification of lysine residues with trinitrophenyl (TNP) groups was found to abolish streptavidin accumulation in the kidney and led to specific and long-term accumulation in the liver [32].

Ouchi et al. [33] reported that the complexes prepared by mixing Bio-TEG-Gal conjugate and fluorescein isothiocyanate (FITC)-labeled Av (feed molar ratio 4 : 1) and mixing Biotin-triethyleneglycol-galactose (Bio-TEG-Gal) conjugate, Bio-TEG-TAMRA conjugate, and FITC-labeled Av are internalized into the hepatoma cells through a receptor-mediated endocytosis mechanism. Similarly, Mamede et al. [34] reported that the avidin-biotin system seems to have potential as a carrier of oligo-DNA to the liver. Zeng et al. [35] reported the synthesis of disulfide-containing polyethylenimine (PEI-SS) from low molecular weight branched PEI and cystamine bisacrylamide (CBA) and then grafted with biotin. The obtained biotinylated PEI-SS was bioconjugated with avidin via the biotin-avidin interaction to form a novel gene vector, biotinylated PEI-SS/avidin bio conjugate (ABP-SS). The results confirmed that ABP-SS contributes to more cellular uptake of complexes in HepG2 cells. Recently, Marysael et al. [36], as an alternative to directly targeting of necrotic tissue using hypericin, synthesized a conjugate of hypericin to biotin for use in a pretargeting approach. Hypericin was conjugated to biotin-ethylenediamine in a straightforward coupling method using n-hydroxysuccinimide and dicyclohexylcarbodiimide. It was concluded from analysis of autoradiography images which show a higher accumulation of I-avidin in pretargeted compared to non-targeted tissue.

#### 4.1.4. Coupling of Targeting Moieties before Nanocarriers Formulation

Another efficient method for the introduction of targeting moieties consists of coupling the selected molecule at one end of a lipid or a polymer. Such strategy can be interesting because the coupling chemistry is realized on lipid or polymer. Moreover, a better control of amount of targeting moieties on nanocarrier surface can be reached, in theory, by introducing a well-defined mol% of targeting moiety bearing a lipid or polymer in the formulation. In contrast to other methods, firstly the targeting moiety is attached to polymer at one of its ends and after that the drug is encapsulated in polymer coupled with ligand molecule. In this method, the chances of leakage of drug molecule is very less as ligand is coupled to polymer before the encapsulation process. By reviewing various literatures, galactose has been used for selective delivery of various drugs encapsulated in nanoparticles using different polymers. Wang et al. [37] prepared liposomes encapsulating doxorubicin and found that doxorubicin loaded galactosylated liposomes presented a high liver accumulation in comparison to doxorubicin loaded conventional liposomes with a loading efficiency of more than 95%. Such high loading efficiency was attributed to positive charge and retention capacity of doxorubicin. Furthermore, the result of intrahepatic distribution and competitive inhibition study also confirmed that galactose residues of doxorubicin loaded galactosylated liposomes could be recognized by ASGP-R on the surface of parenchymal cells leading to high liver accumulation of such targeted liposomes. Jeong et al. [38] synthesized the poly(gamma-benzyl L-glutamate) (PBLG)/poly(ethylene glycol) (PEG) diblock copolymer endcapped with galactose moiety and characterized for study of liver specific targeting and concluded that HepG2 cells with ASGP-R are more sensitive to TX-loaded nanoparticles than free TX, whereas P388 cells, murine leukemia cell line, and SK-Hep 01, human hepatoma cell line, without ASGP-R are less sensitive to TX-loaded nanoparticles than free TX, suggesting that specific interaction between HepG2 cells and galactose moiety of the nanoparticles occurred.

Hattori et al. [39] investigated the potency of the mannosylated cationic liposomes (Man liposomes) and suggested that the targeted delivery of DNA vaccine by Man liposomes is a potent vaccination method for DNA vaccine therapy. Opanasopit et al. [40] reported that muramyl dipeptide (MDP) can be selectively targeted to liver non-parenchymal cell (NPC), including Kupffer cell (KC) using mannosylated-liposomes. Tian et al. [41] firstly modified the chitosan polymer with glycyrrhetinic acid and then prepared the nanoparticles of BSA and concluded that BSA could be entrapped into the nanoparticles with the drug-loading ratio of 26.3% and the encapsulation efficiency of 81.5%. A sustained release over 11-day period was observed in pH 7.4 *in vitro*. Recently, Tian et al. [42] showed that the CTS/PEG-GA nanoparticles were remarkably targeted to the liver and maintained high concentration of drug for prolonged period of time.

## 5. Novel Materials/Ligands Used for Liver Targeting

Different hepatic diseases involve different cells (namely, hepatocytes, Kupffer cell, hepatic stellate cell and sinusoidal endothelial cells, hepatic carcinoma cell, etc.). Hence, it is important to design or select proper materials to target these different diseased cells. In this section, various novel materials used to enhance the targeting efficiency of drug delivery systems to receptors in the liver have been discussed with appropriate cases.

### 5.1. Hepatic Parenchymal Cell Targeting Materials

#### 5.1.1. Asialoglycoprotein Receptor (ASGP-R) Targeting Materials

This receptor is responsible for the clearance of glycoproteins with desialylated galactose or acetylgalactosamine residues from the circulation by receptor-mediated endocytosis.

*Galactose Ligand.* Galactosylated surface is an attractive substrate for hepatocyte culture because of the specific interaction between the galactose ligand and the asialoglycoprotein receptor on hepatocytes. The density of galactose is one of the important parameters for the hepatocyte attachment as it is a major determinant of the hepatocyte attachment, morphology, and functions, as reported by Ashwell and Harford [43]; a multisubunit receptor of hepatocytes is responsible for binding galactose residues on desialylated glycoproteins and triantennary molecules that bind to the lectin with higher affinity than oligosaccharides lacking a third branch [44].

Kobayashi et al. [45] investigated the effects of galactose densities in the galactose carrying PS derivative on morphology, differentiation, and proliferation of hepatocytes. The results indicated that hepatocytes exhibited lower 3H-thymidine uptake under the round morphology on the higher galactose density and higher 3H-thymidine uptake under the spread morphology on the lower galactose density. Ise et al. reported that hepatocytes attached to galactose carrying PS below 20 ng/mL coating density expressed low levels of ASGP-R and exhibited higher proliferative capacities than above 50 ng/mL coating density [46].

The galactose ligand-ASGP-R interaction is not only influenced by the ligand density on the ECM but also by the spatial orientation of the ligand. Cho et al. [47] investigated the effect of galactose orientation on the attachment of hepatocyte to galactose carrying PS surface prepared by the Langmuir-Blodgett (LB) technique. It was found that the hepatocytes cultured on LB surface of the polymer even at the low galactose concentration could well recognize galactose moieties of the polymer owing to the galactose orientation, suggesting that the spatial microdistribution of the galactose in the ECM is important for the regulation of the cell adhesion.

*Lactoferrin (Lf).* It is a mammalian cationic iron binding glycoprotein belonging to the transferrin (Tf) family [48], recently became increasingly attractive because of its multifunctional and mediating biological activities with Lf receptors (Lf-R) [49, 50]. Formerly Lf-R was successfully utilized as a targeting ligand for brain delivery due to the presence of Lf receptor on blood brain barrier (BBB) [51, 52]. Likewise, many recent studies revealed that lactoferrin could bind to multiple receptors on hepatocytes. For instance, Wei et al. reported Lf-PEGylated liposomes (PLS) as a promising drug delivery system for hepatocellular carcinoma therapy with lower toxicity and enhanced efficacy [53–56]. It has been validated that Lf-R binds to ASGP-R with high affinity [57, 58], implying that Lf-R is a good ligand to ASGP-R binding. With its specific binding, Lf-R has been applied to gene delivery successfully, transferring genetic material to the hepatocytes [59]. These exciting evidences suggested that Lf-R might be a promising candidate for hepatocellular carcinoma targeting due to its high affinity for ASGP-R, and developing a hepatic carcinoma targeting drug delivery system employing the ASGP-R targeting ability of Lf-R is highly possible. 

*Lacto Bionic Acid (LA) Ligand.* Kamruzzaman Selim et al. [60] synthesized super paramagnetic magnetite nanoparticles which were surface modified with lactobionic acid (LA) to improve their intracellular uptake and ability to target hepatocytes and reported that LA-modified magnetite nanoparticles have a great potential to be used as contrast agent for liver diagnosis. Later in 2009 [61] beta-galactose carrying lactobionic acid (LA) was conjugated on the surface of mercaptoacetic acid coated cadmium sulfide nanoparticles (CSNPs) to ensure specific recognition of liver cells (hepatocytes) and to enhance biocompatibility and found that the uptake amount of Lactobionic Acid-immobilized CSNPs into hepatocytes was higher than that of CSNPs and Maltotrionic acid-CSNPs. 

*Asialofetuin (AF) Ligand.* AF, a natural ligand, is a glycoprotein that possesses three asparagine-linked triantennary complex carbohydrate chains with terminal LacNAc (N-acetyllactosamine) residues. The expressed protein displays affinity to hepatocyte ASGP-R and is endocytosized by the cells. Its receptor dissociation constant is 200-fold lower than the glycoproteins with biantennary N-linked oligosaccharide chains. Therefore, it has been used as a competitive inhibitor to other polysaccharides that also have affinity to the receptors. Díez et al. [62] synthesized cationic nanoparticles using AF as ligand and concluded that targeted-NP2 particles showed a 5- and 12-fold higher transfection activity in the liver compared to non-targeted (plain) complexes. 

*Soybean-Derived SG Ligand.* Soybean-derived SG is a residue extracted from soybeans. Maitani et al. [63] investigated the interaction of liposomes surface-modified with soybean-derived sterylglucoside (SG) (SG-liposomes) with HepG2 cells and concluded that SG-liposomes are potentially useful drug carriers to target the liver, because the glucose residue may work as a kind of ligand for ASGP-R. Qi et al. [64] studied hepatocyte-specific targeting technology by modifying cationic liposomes with soybean sterylglucoside (SG) and polyethylene glycol and found that C/SG/PEG-liposomes mediated gene transfer to the liver was an effective gene-delivery method for hepatocytes-specific targeting, which appears to have a potential for gene therapy of HBV infections. Later on, Shi et al. [65] constructed a liposomal liver targeting delivery system by adding soybean-derived sterylglucoside (SG) to the cationic liposomes and concluded that SG/Brij-35 modified cationic liposomes are potentially useful drug carrier to the liver.

#### 5.1.2. Glycyrrhetinic Acid (GA) Receptor Targeting Materials

Glycyrrhizin (GL) and glycyrrhetinic acid (GA) are the main bioactive compounds of licorice and are widely used in medicine for the treatment of many pathologies [16, 17], such as antiinflammatory, antigastric, antihepatitis, antiallergic and antihepatotoxic effects. Negishi et al. [66] showed that there are specific binding sites for GL and GA on the cellular membrane of rat hepatocytes and confirm that the number of binding sites for GA is much more than that for GL. Recently, Wolfrum et al. [19] prepared chitosan nanoparticles modified with glycyrrhizin (CTS-NPs-GL) and confirmed that CTS-NPs-GL preferentially accumulated in the rat hepatocytes by a ligand receptor interaction. Akinc et al. [20] also found that the cellular uptake of liposomes modified with glycyrrhetinic acid by rat hepatocytes was 3.3-fold higher than that of unmodified ones. Although the liver cell targeting ability of the GA-modified polymers has been confirmed *in vitro*, there are no reports on the distribution of GA-modified materials *in vivo* and on the presence of GA receptors on human hepatic cells. Therefore, it is necessary to investigate the distribution of GA modified carriers *in vivo* and their affinity for human liver cells. These studies would be of immense importance for the development of GA mediated liver-targeted drug delivery. 

#### 5.1.3. Bile Acid Receptor Targeting Materials

Bile acids and bile acid receptors are therapeutic targets in the development of drugs for the treatment of cholestatic and fatty liver diseases. Clerc et al. [67] determine the effect of exogenous unesterified cholesterol provided in either artificial liposomes or LDL on bile salt and they concluded that taurocholate increased the exchange of cholesterol between liposomes or LDL and hepatocyte membranes. Pütz et al. [68] examine the possibility of targeting liposomes to hepatocytes via bile salts; the bile salt lithocholyltaurine was covalently linked to a phospholipid and concluded that the attachment of bile salts to the surface of hepatocytes opens up promising possibilities for hepatocyte-specific drug delivery.

### 5.2. Hepatic Non-Parenchymal Cell Targeting Materials

#### 5.2.1. Mannose Receptor Targeting Materials

Mannose receptors are known to contribute to the defense mechanism of mammals by endocytosis or phagocytosis of terminal mannose bearing exogenous materials. Mannose, a sugar monomer of hexose, has several important biological roles, including the glycosylation of proteins. Unlike galactose interactions with asialoglycoprotein receptors on parenchymal liver cells, D-mannose is recognized by mannose receptor generally present on non-parenchymal liver cells such as Kupffer cells, macrophages resident in the liver. A mannose 6-phosphate binding protein with a subunit and molecular size of 215,000 has been isolated from bovine liver. The expression of mannose-6-phosphate on rat hepatic stellate cell is increased during liver fibrosis [69]. 

Jayasree et al. [70] has prepared mannosylated chitosan-zinc sulphide nanocrystals and reported that prepared nanobioconjugates through simple aqueous chemistry possessed high colloidal stability and strong fluorescence emission at *∼*600 nm. Characterization using X-ray diffraction, dynamic light scattering, scanning electron microscope, atomic force microscopy, and Fourier transformed infrared spectroscopy revealed that the bioconjugated particles were appropriately functionalized and stable, with an average size of 150 nm. Bioconjugation with mannose provided specificity and targeted cellular labelling characteristics as demonstrated using KB cells which overexpress mannose receptors on their surface.

Rieger et al. [71] reported biodegradable polymeric nanoparticles presented with mannose residue at their surface and their interaction with lectins. The study concluded that prepared nanoparticles are expected to be specifically recognized by mannose receptors which are highly expressed in cells of the immune system. The targeting properties of these carrier systems combined with their potential adjuvant effects due to their size in the range of 200–300 nm make them attractive candidates as vaccine delivery systems.

Kawakami et al. [72] synthesized novel mannosylated cholesterol derivative, cholesten-5-yloxy-N-(4-((1-imino-2-beta-D-thiomannosyl-ethyl)amino)butyl) formamide (Man-C4-Chol), in order to perform mannose receptor mediated gene transfer with liposomes. The results reported by them suggest that plasmid DNA complexed with mannosylated liposomes exhibits high transfection activity due to recognition by mannose receptors both *in vitro* and *in vivo*.

#### 5.2.2. Hepatic Stellate Cell (HSC) Targeting Materials

HSCs play a central role in the progression of liver fibrosis. Muriel et al. [73] investigate a suitable model of fibrosis, in which spontaneous reversion was minimal, in order to study the ability of silymarin, silibinin, colchicine, and trimethylcolchicinic acid (TMCA) to reverse it and the model reported by them was proved to be an excellent tool to study the ability of drugs to reverse fibrosis. Suojanen et al. [74] studied the tumour growth and lymphatic micrometastatic by use of HSC-3-cell xenografted athymic nude mice and concluded that peptide gelatinase inhibitors are effective in inhibiting primary tumor growth but alone they do not prevent the spread of carcinoma cells.  Table 2 provides compilation of different targeting ligands employed for enhanced liver targeting.

## 6. Targeting Strategies for Different Constituent Cells of Liver and Their Implications

The approaches to drug targeting described so far are not universal. Direct administration of a drug into an affected organ or tissue may be technically difficult. Often, the affected areas do not differ much from the normal tissues in terms of vascular permeability, temperature, and local pH value. Magnetic drug delivery also has limitations connected with blood flow rate in the target. The most natural and universal way to impart the affinity toward the target to a nonspecific drug is the binding of this drug with another molecule (targeting moiety) capable of specific recognition and binding at the target site. The following substances can be used as targeting moieties: antibodies and their fragments, lectins, other proteins, lipoproteins, hormones, charged molecules, mono-, oligo- and polysaccharides, and some low molecular weight ligand such as sugars, folic acids, and peptides. The parameters that determine the efficacy of drug targeting include the size of target, blood flow through the target, number of binding sites for the targeted drug/drug carrier within the target, number and affinity of targeting moieties on a drug molecule, and multipoint interaction of a drug/drug carrier with the target [88]. Targeting moiety should be selected by keeping the following points in mind: (i) reaction between carrier and moiety has to be simple, fast, efficient, and reproducible, (ii) coupling method has to yield to stable and nontoxic bond, (iii) target recognition and binding efficiency of the coupled molecule have to be maintained, (iv) targeted nanocarriers have to be stable enough to present a circulation half-life allowing them to reach and interact with their site of action, and finally (v) both the drug loading efficiency and the drug release profile do not have to be significantly changed by targeting moieties coupling reactions.

### 6.1. Liver Cell Specific Targeting of Therapeutics

Carrier molecules are designed for selective cellular uptake, taking advantage of specific receptors or binding sites present on the surface membrane of the target cell (Table 1). In various literatures, hepatocytes targeting is synonymous for liver targeting and total liver uptake of a compound is measured without proper identification of the cell type that actually takes up the drug. Although hepatocytes represent more than 80% of the total number of resident hepatic cells, uptake in other cell types like Kupffer cells may occur as well and high uptake of viruses, antibodies, or other biological compounds into these cells often leads to complete degradation of such compounds, which in some cases destroys their therapeutic activity [13]. Therefore, for specific cell, there should be specific delivery system and ligand for targeting. There may be five different cells types present in liver for active targeting of drug, namely, (i) hepatocytes, (ii) Kupffer and sinusoidal endothelial cells, (iii) hepatic stellate cells (HSC), (iv) bile duct epithelial cells, and (v) hepatocellular carcinoma cells. Table 3 provides details of targeting approaches of drugs for different constituent cells of liver.

#### 6.1.1. Hepatocytes

Hepatocytes are generally the affected site in various liver diseases like viral hepatitis (hepatitis A, B, or C), alcohol-induced steatohepatitis (ASH), nonalcohol-induced steatohepatitis (NASH), and some genetic diseases like Wilson's disease, hemochromatosis, *α*-1 antitrypsin deficiency, and several other metabolic disorders. In order to reduce the side effect and enhance the therapeutic effect of drugs, many methods for hepatocytes selective drug targeting have been used in the past decades. The most prevalent and the effective strategies for targeting hepatocytes are mentioned below.

Asialoglycoprotein receptors (ASGP-R) are exclusively found in hepatocytes located at the basolateral membrane and therefore are in direct contact with the bloodstream. The human ASGP-R is a heterooligomer that is composed of two homologous subunits (46 and 50 kDa). ASGP-R recognizes with high affinity (KD in the nanomolar range) tri- and tetra-antennary N-linked sugar side chains with terminal galactose residues [109]. Therefore, galactose residue [17, 110] or lactose moieties [111] act as ligand and are coupled to proteins polymers or incorporated into the outer layer of delivery system by one of the methods described above [16]. Glycoproteins with such glycosylation patterns are rapidly endocytosed by the ASGP-R via clathrin-coated pits and vesicles. In a similar context, Shinoda et al. [112] investigate specific interaction between galactose branched cyclodextrins (gal-CyDs) and hepatocytes *in vitro* and concluded that enzymatically synthesized gal-CyDs have specific interaction with the hepatocytes and may be useful as a drug targeting carrier to hepatocytes. But when efficient delivery of liposomes to hepatocytes was performed by targeting the galactose receptor on the surface of hepatocytes by lactosylceramide and asialofetuin then it was found that lactosylceramide containing vesicles distributed 48% in hepatocytes and 27% in non-parenchymal cells [113]. It has been shown that a galactose receptor is also located on Kupffer cells and involved in endocytosis or phagocytosis of particles with galactosyl residues [69]. These receptors are not evenly distributed on the surface of Kupffer cells but clustered for the uptake of particles [70]. However, the galactose receptors on hepatocytes are evenly distributed [71] and can uptake ligand of up to 8 nm in diameter [72]. Thus, galactose moiety to target asialoglycoprotein receptor may not be a good target for the selective delivery of liposomes to hepatocytes. Therefore, the discovery of new ligands for liver targeting, instead of the use of the conventional ligands, is very important. Another ligand for hepatocytes was investigated by Lin et al. [114] in which chitosan nanoparticles surface was modified with glycyrrhizin (CS-NPs-GL) as new hepatocyte-targeted delivery vehicles and concluded that CS-NPs-GL could be a promising hepatocyte-targeted delivery carrier. But Negishi et al. [66] showed that the number of binding sites for glycyrrhetinic acid (GA) is much more than that for GL. Therefore, Tion et al. [115] prepared glycyrrhetinic acid-modified chitosan/poly(ethylene glycol) nanoparticles for liver-targeted delivery and found that the cellular uptake of nanoparticles modified with glycyrrhetinic acid by rat hepatocytes was 19-fold higher than that of unmodified ones. In fact delivery of NDDS (like liposomes, niosomes, nanoparticles, and phytosomes) and proteins to the hepatocytes using ASGP-R as a target receptor was one of the first options for the cell specific delivery to the liver cells although this has not led to any clinical application yet.

#### 6.1.2. Kupffer and Sinusoidal Endothelial Cells

Kupffer and sinusoidal endothelial cells are localized within the space of disse in close vicinity of the hepatocytes [116]. Kupffer cells, the resident liver macrophages, have long been considered as scavenger cells responsible for removing particulate material from the portal circulation. However, evidence derived mostly from animal models indicates that Kupffer cells may be implicated in the pathogenesis of various liver diseases including viral hepatitis, steatohepatitis, alcoholic liver disease, intrahepatic cholestasis, and activation or rejection of the liver during liver transplantation and liver fibrosis [117]. Both the Kupffer and sinusoidal endothelial cells share many characteristics that can be relevant for the enhanced uptake of drug delivery systems by these cells. They are endowed with a high phagocytic capacity and are as such an intrinsic part of the reticuloendothelial system (RES). This system plays an essential role in the regulation of the host defence system and because of this, many foreign particles that accumulate in these two cell types [118]. Several of these diseases are treated with systemic immunosuppressive agents, but there are many arguments that favour a local downregulation of inflammatory processes within the liver rather than inducing systemic immunosuppressive effects [119]. Accumulation of therapeutic compounds in Kupffer and endothelial cells can either be nonspecific or specific via designated receptors. Drug delivery systems like liposomes, micelles and viral particles end up in these cells via nonspecific uptake mechanisms due to their largest phagocytic activity [120].

In addition, uptake in Kupffer and endothelial cells can also be mediated by specific receptors. These cells bind to negatively charged molecules via scavenger receptors that are abundantly expressed on their membrane [121, 122]; for example, coupling of compound to lysine groups within the albumin molecule removes positive charges from the molecule which creates a compound with a net negative charge that may serve as a ligand for scavenger receptors [123, 124].

Targeting to Kupffer cell is directed through mannose receptor using sugar moieties (like mannose and fucose) which are coupled to delivery system while targeting to sinusoidal endothelial cell is possible using hyaluron receptor as the target receptor [5, 6]. Yamashita et al. investigated that when liposomes surface was modified by cetylmannoside (Man) then it could be useful for targeting to Kupffer cells [125]. Melgert et al. investigated that when dexamethasone was coupled to mannosylated albumin, it is selectively delivered to the Kupffer cell [126].

#### 6.1.3. Hepatic Stellate Cells (HSC)

Hepatic stellate cells (HSC) play a crucial role in the development of liver fibrosis because of their prominent role in extracellular matrix production, regulation of vascular tone, and production of inflammatory mediators such as transforming growth factor-b (TGF-b) and platelet-derived growth factor (PDGF). During fibrosis, in particular, these three processes are dearranged. At a certain point in the whole process, the HSC perpetuate the fibrogenesis by creating several autocrine loops, thus maintaining the process even without contribution of the other cell types. Therefore, these cells are major target for antifibrotic drugs [127–131]. The first target receptor chosen was the mannose-6-phosphate (M6P)/insulin-like growth factor II (M6P/IGFII) receptor, because it was reported to be highly unregulated on the cell membranes of activated HSC. HSA was modified with the sugar mannose-6-phosphate [132]. More importantly, the major part of the hepatic content was found in the HSC. Mannose-6-phosphate hepatic stellate cell (M6P-HSA) bound in particular to the activated HSC and a rapid internalization of the protein occurred via a receptor-mediated endocytotic route. Greupink et al. [133] showed that targeted delivery of coupled mycophenolic acid to the HSC-selective drug carrier mannose-6-phosphate modified human serum albumin results in a decrease in HSC activation, making it the first drug that is successfully delivered to this cell type. Adrian et al. [134] confirmed that M6P-HSA-liposomes can be efficiently targeted to non-parenchymal cells, including HSC. In addition, two other HSC selective carriers were found. Instead of the derivatisation of albumin with specific sugars, albumin was now modified with cyclic peptide moieties (minimized proteins) that represented the binding domains of cytokines/growth factors responsible for binding to the activated HSC; for example, pentoxifylline is a promising drug that have been benefited from drug targeting strategies [7, 135–138].

Not only drugs but genetic materials are also of interest to be specifically targeted to HSC. Gene therapy is an elegant way to correct genetic deficiencies or to induce the production of essential proteins in a certain cell type. Adenoviral or lipid based nonviral vectors are alternatives to deliver genes and antisense material to cells [139–144]. A few reports on gene delivery to the cirrhotic liver or to HSC have appeared in the past few years, mostly using adenoviral mediated transduction methods. Yu et al. [145] reported a hepatic delivery of neuronal nitric oxide synthase (NOS) which resulted in a reduction of the intrahepatic resistance and portal pressure in *in vivo* models of liver fibrosis. Qi et al. [146] delivered a dominant-negative type II transforming growth factor-b receptor gene to the liver and found a blocking of transforming growth factor-b, which attenuated the development of liver fibrosis. Other examples of gene delivery in the context of liver fibrosis are the hepatic delivery of telomerase RNA by Rudolph et al. [147] and of urokinase-type plasminogen activator by Salgado et al. [148] and many more but without clinical usefulness.

#### 6.1.4. Bile Duct Epithelial Cells

Bile duct epithelial cells play a key role in the pathogenesis of several hepatic diseases [149]. In primary biliary cirrhosis, these cells are the target of an autoimmune disease leading to the destruction of small intrahepatic bile ducts. The etiology is not yet clear, but the chronic inflammatory reaction around bile duct epithelial cells ultimately leads to irreversible cirrhosis and end-stage of liver failure.

Cell specific delivery to this cell type is still in its infancy. Until very recently, no drug carrier to this cell type was described. Oja et al. reported the expression of secretin receptors on cells of the biliary tract [150]. This receptor appears to be present on normal bile duct epithelial cells and ductules and they suggested that this receptor may be used for targeting to cholangiocarcinomas for therapy or diagnosis of this disease because of very high receptor expression on these carcinoma cells [151].

#### 6.1.5. Hepatocellular Carcinoma Cells

Hepatocellular carcinoma (HCC) is one of the most common malignant tumors which can result from several liver diseases such as hepatitis B and C infections, metabolic liver diseases, and nonalcoholic fatty liver diseases [152]. Malignant transformation of hepatocytes induced by viral factors and several other mediators in the chronically inflamed area leads to hepatocarcinogenesis. HCC is the sixth most common cancer (in male) and the third leading cause of cancer associated deaths in the world [153, 154]. In well-differentiated forms of HCC, hepatocytes express the asialoglycoprotein (ASPG) receptor [155]. Many drug delivery systems have already been developed to deliver drugs to this receptor using lactosaminated or galactosamine substituted drug carriers. In particular, polymers have been applied for the purpose of drug delivery to HCC [156–158] but modified albumins have also been explored [159, 160]. The ASGP-receptor has also been employed as a target receptor for the delivery of genes with antineoplastic effects to the hepatocytes by making complexes of plasmids and polymers coupled to ASGP-receptor binding ligands [161]. Also other drug delivery systems such as cationic liposomes, virosomes, and adenoviral vectors have been exploited for the delivery of anticancer drugs and genes in HCC. Cheng et al. [162] synthesized GC/5-FU nanoparticles by combining galactosylated chitosan (GC) material with 5-FU, and tested its effect on liver cancer *in vitro* and *in vivo.* They conclude that sustained releases of GC/5-FU nanoparticles are more effective at targeting hepatic cancer cells than 5-FU monotherapy in the mouse orthotropic liver cancer mouse model. In the same year, Li et al. [163] concluded that the encapsulation of tetrandrine (Tet) and TX into nanoparticles retain the synergistic anticancer efficiency of Tet and TX against mice hepatoma H22 cells. Other researchers, Zhou et al. [164], synthesized N-lactosyl-dioleoylphosphatidylethanolamine (Lac-DOPE) and evaluated as a liver-specific targeting ligand via ASGP-R receptors for liposomal delivery of doxorubicin and reported that lactosylated liposomes are promising drug delivery vehicles for hepatocellular carcinoma. Recently, Zhang et al. [165] reported that mixed copolymer nanoparticles (NPs), self-assembled from *β*-cyclodextrin-grafted hyperbranched polyglycerol (HPG-g-CD) and lactobionic acid (LA)-grafted hyperbranched polyglycerol (HPG-g-LA), were utilized as carriers for hydrophobic antitumor drug, TX, resulting in enhanced hepatocellular-carcinoma uptake of these nanoparticles. Therefore, it was concluded that mixed copolymer NPs are efficient nanocarriers for hepatoma-targeted delivery of potent antitumor drugs. 

## 7. Expert Comments

As per the report given by the WHO, approximately 1 in 12 persons worldwide or some 500 million people are living with chronic liver disease. It is forecasted that liver diseases may become a higher ranked cause of death in coming decades because of its complex pathogenesis which involves a variety of cells (parenchymal and non-parenchymal). The literature expressed that more than 90% of therapeutic drug is uptaken by normal tissues, whereas only 2%–5% is uptaken by diseased cells and moreover the current available therapy for liver diseases lacks adequate specificity and efficacy. To limit the severe side effects of conventional therapy, targeted delivery systems have shown an attractive prospect and opened a door for the treatment of chronic liver diseases. Hence, to increase the efficacy of drugs and decreasing their toxic side effects, encapsulation of drug in a carrier system is a common approach to achieve passive targeting. Nevertheless, passive targeting does not always lead to effective drug accumulation in a specific tissue or organ. Therefore, to increase the specificity of interaction between drug delivery system (DDS) and target cells or tissues as well as to increase the amount of drug delivery to the desired site of action, active targeting is needed. Such active targeting can be obtained by coupling a targeting moiety to the DDS, providing a selective and quantitative accumulation of the DDS at the target site. In this context, the concept of active liver targeting is undergoing clinical trial study, for example, doxorubicin coupled with magnet (MTC-DOX) is expected to target liver tumors directly. One of the recent approaches in which the drug is directly bound to a targeting moiety, for example, when norcantharidin directly bound to galactose group resulted in higher entrapment efficiency and low toxicity, still needs more focus to explore. Whatever the way selected for coupling targeting moiety to a DDS, the reaction has to be simple, fast, efficient, and reproducible. Both the drug loading efficiency and the drug release profile do not have to be significantly changed by targeting moieties coupling reactions. Furthermore, targeted DDS have to be nontoxic and stable enough to present a sufficiently long circulation half-life allowing them to reach and interact at their site of action. 

It is also necessary to explore whether fabricated delivery system could also show the same targeting efficiency in humans with chronic liver diseases as in animal models. Efficient drug targeting to liver has certain limitations for medical applications: efficacy, safety, and cost as well as regulatory concerns. 

Efficacy and safety of the developed formulations remain the prime goals with which these are developed. It is essential that the drug delivery vehicle should deliver the drug payload at its intended site of action at a required rate. An ideal carrier should be inert and devoid of any harmful effects. Another major restraint in designing the targeted delivery system is the high cost which makes productivity more difficult and the reduced ability to adjust the dosages. This could be overcome by encouraging the building of strong partnerships at the national and international level among academic and industrial partners with multidisciplinary expertise. Another hurdle is receiving the approval from drug regulatory authorities of respective countries such as Food and Drug Administration (FDA) in the USA, European Agency for the Evaluation of Medicinal products (EMEA) in Europe, and Pharmaceutical and Medical Device Agency, KIKO (PMDA, KIKO) in Japan due to long-term clinical trials owning to the lack of surrogate parameters to measure the effect of treatment. Duration of clinical trial can be reduced by coupling the radiolabel substance or fluorescent dye to DDS. This dual approach, that is, the use of the same carrier for imaging and treatment, will speed up the clinical trial study as well as approval from regulatory authorities. 

Therefore, a lot of work remains to be done for the efficient targeting of drug to liver. Next improvements will certainly come from the introduction of new materials including stimuli-responsive polymers to elicit the challenge of targeting the drug to its specific site of action, to retain it for the desired duration, and to release it according to the correct time schedule. 

## 8. Conclusion

The pathogenesis of liver diseases involves a variety of cells which makes the delivery of drug complicated. The most important aspects to improve the treatment via hepatoprotective drug are the design and synthesis of appropriate polymeric material to target specific cells of liver. Ingenious studies are required in coupling and selection of targeting moiety so that they could be translated from the bench research to the bedside. We have reviewed various aspects of selection of ligands and their coupling to drug/polymer which would potentially target parenchymal/non-parenchymal liver cells. The pharmacokinetic behaviour and physicochemical factors related with delivery systems have been considered to be primarily responsible for the improved targeting and therapeutic effectiveness; therefore, dealing with these factors during formulation development could lead to more promising treatments for acute and/or chronic liver diseases. These investigations require thorough inspection as well as innovative approaches to bring them into the global market at affordable price. 

## Figures and Tables

**Table 1:** Receptors present on various hepatic cell and may be used for drug targeting [25].

Hepatocytes	Kupffer cells	Endothelial cell	Hepatic stellate cells
Asialoglycoprotein receptor (ASGP-R)	Mannose/N-acetyl glucose amine R	Mannose/N-acetyl glucose amine R	M6P/IGF II R
HDL-R	Galactose particle R	Scavenger R (Class A1 and A11)	*α*_2_ macroglobulin R
LDL-R	Galactose specific R	Fc R immune complexes	Ferritin R
IgA-R	Fc R (immune complexes, opsonized material)	Matrix compound (hyaluronan fibronectin, denatured collagen PIIINP)	Uroplasminogen R
Scavenger R (Class BI)	Scavenger R (Class AI, BI, BII, MARCO CD36 and macrosialin)		Thrombin R
Transferrin R	LDL R matrix compounds (fibronectin)		RBP R matrix compounds (intregrin, collagen type VI, fibronectin CD_44_)
Insulin R	Complement R (C3b and C1q) LPS R *α*_2_ macroglobulin R		

*R: Receptor.

**Table 2:** Different ligand-based liver targeting approaches and conclusions.

Drug	Ligand	Conclusion	References
Doxorubicin	Galactosamine	GAL-DOX-AN is more effective in killing HepG2 cell than DOX-AN	[75]

Sugar charge-modified albumins	Lactose, mannose	Study demonstrates that cell-specific delivery of sugar- and charge-modified albumins in fibrotic livers is possible by coupling drugs to lactose and mannose	[76]

5-Iodo 2′-deoxyuridine 5′-monophosphate	Lactose	Bioavailability of 5-iodo 2′-deoxyuridine 5′-monophosphate to the parenchymal liver cell is dramatically enhanced as a result of the conjugation of the antiviral drugs to lactosylated poly-L-lysine	[31, 77]

Dextran	Permanent magnets and calcein as a fluorescent marker	Dextran magnetite (DM)-incorporated thermosensitive liposomes would be useful in future cancer treatment by magnetic targeting combined with drug release in response to hyperthermia	[78]

Tyr^3^-octreotide (TOC), a somatostatin analogue	*N*-palmitoyl cysteinyl moiety	Tyr^3^-octreotide (TOC), a somatostatin analogue shows enhanced therapeutic efficacy due to the liver-targeting effect when coupled with *N*-palmitoyl cysteinyl moiety.	[79]

Methotrexate conjugated with bovine serum albumin (BSA)	Galactose	Galactosylation of the carrier protein BSA significantly enhanced the hepatocytes uptake and liver targetability of MTX	[80]

Prednisone acetate conjugated with polycaprolactone-g-dextran polymer	Galactose and fluorescein isothiocyanate	*In vivo* study indicated potential of prednisone acetate loaded galactosylated micelles in liver targeting	[81]

Lactobionic conjugated with chitosan	Azide (CHI-Az) or alkyne (CHI-Alk) groups	Lactobionic acid was conjugated with (CHI-Az/CHI-Alk)-coated particles and the particles exhibited hepatoma cell (HepG2) targeting behavior	[82]

Doxorubicin	Soybean-derived sterylglucoside (SG)	SG liposomes are potentially useful drug carriers to the liver, because the glucose residue may work as a kind of ligand for asialoglycoprotein receptor (ASGP-R) on hepatocytes	[63]

Primaquine phosphate conjugated with polypropyleneimine (PPI) dendrimers	Coated peripherally with galactose	The galactose coating of PPI dendrimers can make the PPI systems more effective and suitable for targeted delivery of primaquine phosphate to liver	[83]

Paclitaxel conjugated with PLGA	Galactose	Paclitaxel loaded galactosylated PGA-PLA nanoparticles were mainly accumulated at the tumor site and the liver	[84]

Rhein	Vinegar-baked Radix Bupleuri	Co-administration of rhein with VBRB efficient for liver targeting	[85]

Streptavidin	Trinitrophenyl (TNP) groups	The modified proteins could target high doses of chemotherapeutic drugs (CDDP and 5-fluorouridine) to the liver through biotinyl dextran-derived carriers	[86]

5-Fluoro 29-deoxyuridine conjugated with lactosaminated poly-L-lysine	Lactose	Poly-L-lysine-5-fluoro-2′-deoxyuridine enters into HepG2 cells through the asialoglycoprotein receptor and, after intracellular penetration, releases the drug in a pharmacologically active form	[87]

**Table 3:** Cell specific hepatic targeting of different drugs.

Type of cell/receptor	Drug	Further remarks	References
Hepatocytes and asialoglycoprotein receptor	Iododeoxyuridine (IDU)	By isolating liver cells after injection of the iododeoxyuridine (IDU), it was concluded that hepatic uptake occurred mainly in parenchymal liver cells	[89]

Hepatocytes	Primaquine (PQ)	The prepared emulsion could be developed into a promising delivery system to target PQ into hepatocytes for vivax malaria therapy	[90]

Hepatocytes	5-Fluorouracil (5-FU)	The drug-loaded ZPs* could be efficiently targeted at the liver by intravenous delivery	[91]

Hepatic stellate cell (HSC)	Human serum albumin (HAS) modified with mannose6-phosphate (M6P)	M6P-modified albumins are taken up by HSC in fibrotic livers	[92]

Hepatic stellate cell (HSC)	MicroRNAs	The study shows that there was direct target of miR-181b in HSC-T6 cell	[93]

Hepatic stellate cell (HSC)	Antibody fragment	This antibody fragment may be an effective means to target therapeutics to human hepatic stellate cells	[94]

Hepatic carcinoma cell	Glycyrrhetinic acid-modified poly(ethylene glycol)-b-poly(c-benzyl L-glutamate) (GA-PEG-PBLG) micelles	*In vitro* cell uptake results indicated that the introduction of GA to the micelles significantly increased the affinity for human hepatic carcinoma	[95]

Hepatic carcinoma cells	Ribavirin nanoparticles	The nanoparticles had effective growth inhibitory activity in hepG2 human hepatoma cells	[96]

Hepatic carcinoma cell	Rhodamine B with lactose as ligand	The Lac-micelles will be an effective liver-targeting drug delivery system	[97]

Kupffer cell (nonparenchymal cells)	Cholesten-5-yloxy-*N*-(4-((1-imino-2-b-D-thiomannosylethyl)amino)butyl)formamide (Man-C4-Chol) into small unilamellar liposomes consisting of cholesterol and distearoyl 3 phosphatidylcholine (DSPC)	The results suggest that Man liposomes are effective carriers for targeted delivery of bioactive compounds to liver NPC	[40]

Hepatic stellate cell (HSC)	Pentoxifylline	PTX-neoglycoprotein mannose-6-phosphate-albumin (M6PHSA) employing a novel type of platinum linker, which allows sustained delivery of the drug to HSC in the fibrotic liver	[98]

Hepatocytes	Galactosylated poly(ethylene glycol)-chitosan-graft-polyethylenimine (Gal-PEG-CHI-g-PEI)	Together, these results suggest that Gal-PEG-CHI-g-PEI, which has improved transfection efficiency and hepatocytes specificity both *in vitro* and *in vivo*, may be useful for gene therapy	[99]

Hepatocytes (asialoglycoprotein R)	Vitamin K5 and cytosine arabinoside using poly-L-glutamic acid and carboxymethyl dextran	Effective targeting to hepatocytes	[100]

Hepatic carcinoma cells	Ursodeoxycholic acid (UA) modified protein-lipid nanocomplex	The uptake of UA modified protein attached on the nanoparticles was higher in hepatic carcinoma cells (HepG2 and Bel 7402) than in normal liver cells	[101]

Hepatic carcinoma cell	Human telomerase reverse transcriptase with pegylated immuno-lipopolyplexes	The vector pApoAI-shTERT was able to cause liver-specific and hTERT target-specific cytotoxicity, and utilizing PILP to deliver pApoAI-shTERT is a promising strategy for liver-specific gene therapy	[102]

Nonparenchymal cells	Mannosylated superoxide dismutase (SOD)	Increased delivery of SOD to nonparenchymal cell	[103]

Hepatocytes	Probucol liposomes	Hepatic uptake of liposomes should be mediated by asialoglycoprotein receptors being probucol incorporated in them	[104]

Endothelium cell	Paclitaxel (PTX)-loaded PEGylated PLGA-based nanoparticles grafted with RGD peptide	The targeting of anticancer drug to tumor endothelium by RGD-labeled NP is a promising approach	[105]

Hepatic stellate cells (HSC)	Cyclic Arg-Gly-Asp (RGD) peptides were combined with maleimide-[poly(ethylene glycol)]-1,2-dioleoyl-sn-glycero-3-phosphoethanolamine (MAL-PEG-DOPE) incorporated into stabilized liposomes	Targeted liposomes encapsulating HGF are a promising therapeutic modality in terms of promoting the remission of liver cirrhosis by promoting collagen fiber digestion, inhibiting collagen production and promoting apoptosis of *α*-SMA-positive cells in rats with cirrhosis	[106, 107]

Hepatocytes (asialoglycoprotein receptor)	Super paramagnetic iron oxide (SPIO) nanoparticles	These data underline the potential application of Gal-ASPIO as a targeted ligand for ASPGR-expressing cells *in vivo *	[107]

Hepatic carcinoma cell	Doxorubicin loaded super paramagnetic iron oxide nanoparticles	DOX is a promising candidate for treating liver cancer and monitoring the progress of the cancer using MRI	[108]

*Zein nanoparticles.
